# Electrodermal lability and sensorimotor preparation: effects on reaction time, contingent negative variation, and heart rate

**DOI:** 10.3758/s13415-024-01206-8

**Published:** 2024-08-14

**Authors:** Heinz Zimmer, Fabian Richter

**Affiliations:** 1 Department of Psychology, University of Cologne, 50931 Cologne, Germany; 2 Deutsches Herzzentrum der Charité – Department of Cardiothoracic and Vascular Surgery, 13353 Berlin, Germany; 3 Charité – Universitätsmedizin Berlin, corporate member of Freie Universität Berlin and Humboldt-Universität zu Berlin, Charitéplatz 1, Berlin, 10117 Germany

**Keywords:** Electrodermal lability, Expectancy wave, Heart rate deceleration, Nonspecific electrodermal response frequency, Vigilance

## Abstract

Electrodermal lability is a trait-like measure of spontaneous sympathetic resting activity. In the present study, we addressed whether interindividual differences in this lability have an impact on the reaction time (RT) and on two physiological indicators of a goal-oriented sensorimotor preparation in a long-running, forewarned RT task (S1-S2 paradigm). The two indicators were the brain’s contingent negative variation (CNV) and a heart rate deceleration (HRD). The interindividual differences were determined by counting spontaneous skin conductance fluctuations during a 5-min resting phase and dividing the subjects into two groups: individuals below (stable) and above (labile) the median of these fluctuations. In the task, labile individuals had a shorter RT compared with stable individuals and showed in the final phase of preparation in both physiological indicators the stronger response. Thus, lability-dependent effects in forewarned RT tasks cannot be explained by differences in stimulus-driven or passively controlled processes alone. Rather, goal-oriented, deliberately controlled processes that serve to adequately prepare for an imperative stimulus—the S2 in our paradigm—also must be considered to explain them. Labile individuals not only react faster than stable ones but also intentionally prepare themselves more appropriately for the imperative stimulus. A norepinephrine hypothesis focusing on the tonic activity of the locus coeruleus (LC) is proposed as an explanation for these and other lability-dependent effects. The frequency of spontaneous electrodermal fluctuations at rest may represent a peripheral, noninvasive, and easily measurable indicator of the baseline LC activity during wakefulness.

## Introduction

Measuring electrodermal activity (EDA) on the palm of a hand during a resting phase reveals remarkable interindividual differences in the frequency of relatively brief fluctuations in skin conductance, all of which occur without muscle activity and above all without any apparent cause—i.e., nonspecific or spontaneous. Yet, they resemble induced reactions. Moreover, these interindividual differences remain largely stable over time and across numerous situations, which is why the frequency of these *spontaneous fluctuations* (SF) in skin conductance is attributed a trait-like character and why they are used for classifying individuals (Bari, 2019; Crider, 1993; Crider & Lunn, 1971; Crider et al., 2004; O’Gorman & Horneman, 1979; Schell et al., 1988, 2002). Twin studies have shown that around 50% of its variance is hereditary (Isen et al., 2012; Vaidyanathan et al., 2014). Anomalies in this activity have even been observed in a variety of psychological disorders, including childhood conduct disorder (Herpertz et al., 2001), criminal psychopathy (Raine et al., 1995), and schizophrenia (Schell et al., 2002).

To determine this frequency, one simply needs to search the palmar skin conductance recording for SF, count them, and relate the number to the duration of the resting measurement. Typically, a 5-min resting measurement is used for this purpose. Across individuals, the number of SF per minute can vary from none to more than 15 (Vossel & Zimmer, 1990). Individuals with many SF are traditionally classified as electrodermally labile and individuals with few SF as electrodermally stable (Mundy-Castle & McKiever, 1953). Often, the terms “Labiles” versus “Stabiles” are used for the two characteristic values or categories, whereas "lability" is used for the associated trait variable.

The fascinating thing about electrodermal lability at rest is its ability to predict brain performance in various functional domains. This appears to be the case, because it reflects a brain state that modulates numerous (automatic and controlled) brain processes. Thus, it is of relevance as both a trait and a state variable.

For example, when looking at the electrodermal and cardiac response to an orienting stimulus, an increase in skin conductance and a heart rate deceleration (HRD) is usually observed for a short period of time (Zimmer, 2002; Zimmer & Richter, 2023). The strength of these reactions may covary with the electrodermal resting lability. On average, labile individuals are expected to react more strongly than stable individuals in both cases (Schell et al., 1988). This is because labile individuals react more sensitively to orienting stimuli both sympathetically, as can be seen from the electrodermal response, and parasympathetically, as can be seen from the HRD. The electrodermal resting lability thus reflects a systemic condition that modulates the central nervous regulation of both branches of the autonomic nervous system, although it is itself only of sympathetic origin (for the *peripheral* causal mechanism: Hagbarth et al., 1972; Wallin, 1981).

Although electrodermal lability is an easily measurable variable, it has only been researched to a limited extent. Its psychological significance and central nervous origin are largely unknown. However, it is known that the central nervous system states underlying the manifestations of electrodermal lability affect—in ways that remain to be elucidated—the orienting response (Vossel & Zimmer, 1992; Zimmer & Demmel, 2000), performance in forewarned reaction time paradigms (Wilson, 1987), vigilance performance (Crider, 1979), and the tendency to fall asleep in monotonous situations (Bohlin, 1973). Thus, electrodermal lability may be related to the bottom up (stimulus-driven) and top-down (goal-driven) control of attention as well as to the control of vigilance, wakefulness, and sleep. Typically, labile individuals perform better under such conditions than stable individuals (Crider, 1993).

The present study investigated whether individuals differing in their electrodermal lability also differ in their adaptive, intentionally controlled processes. For this purpose, two electrophysiological indicators of goal-oriented sensorimotor preparation were measured in a long-running, forewarned reaction time paradigm. We measured (a) the *contingent negative variation* (CNV, Walter et al., 1964) in the electroencephalogram (EEG) and (b) the *terminal heart rate deceleration* (tHRD) in the electrocardiogram (ECG).

In forewarned reaction time paradigms (also known as S1-S2 tasks), a first (warning) stimulus (S1) precedes at a constant time interval a second (imperative) stimulus (S2) to which a reaction is to be made. In such a paradigm, preparation for the S2 manifests itself in a shortened reaction time (RT) and in a late or terminal CNV, among other things. Depending on its functional significance, the CNV is divided into an early (initial) CNV—an O-Wave (Loveless & Sanford, 1974) or Negative Afterwave (Rohrbaugh et al., 1976)—and a late (terminal) CNV or Expectancy Wave (Loveless & Sanford, 1974). Unlike the Readiness Potential (Deecke et al., 1969), which is primarily investigated in voluntary movements (Brunia, 1993; Kornhuber & Deecke, 1965; Libet, 1985), the terminal CNV reflects not only motor preparation but also stimulus anticipation (Brunia, 1988; Brunia & Damen, 1988; Damen & Brunia, 1987; van Boxtel & Brunia, 1994). For this reason, it also is referred to as Stimulus Preceding Negativity. Following convention, we prefer the abbreviation CNV or, depending on the context, terminal CNV.

In view of the already known correlation between RT and CNV (Rebert & Tecce, 1973), it is expected (Hypothesis 1) that not only the RT but also this brain wave will show significant effects of lability—provided that the central nervous processes underlying lability have an impact on goal-oriented, adequate preparatory processes.

Recent research also suggests that preparation is a set of processes executed over time (Jennings & van der Molen, 2005) and that a transient slowing of heart rate before S2 indicates inhibitory processes necessary for appropriate task-related preparation (Jennings & van der Molen, 2002). This task-related change in heart rate—the tHRD—might therefore also allow insights into the functioning of the prefrontal attention control system^1^ during preparation (Jennings & van der Molen, 2002, p. 337–340). Considering this and the parallelism between terminal HRD and terminal CNV (Damen & Brunia, 1987), it is expected (Hypothesis 2) that a significant effect of lability will also manifest itself in tHRD.

Our study was therefore inspired by two main questions. Is there evidence for lability effects in both indicators (CNV and tHRD) of goal-oriented preparation? Can these effects help to explain the established relationship (Wilson, 1987; Wilson & Graham, 1989) between lability and RT?

## Methods

### Participants

The study included data from 48 male students (mean age: 25 years, range: 20–36). Inclusion criteria were right-handedness, unrestricted hearing and vision as well as physical and mental health. The participants were assigned to the lability groups (stable – labile) based on their spontaneous electrodermal activity (see participant classification). There were no significant age differences between the two groups. Participants received a compensation of 10 € for their participation. The study was conducted in accordance with the ethical standards of the 1964 Helsinki Declaration and its later amendments. Informed consent was obtained from all participants before the study.

### Study environment and boundary conditions

The study took place within a soundproof and diffusely lit metal cabin in which the room temperature (24 °C) and relative humidity (55%) were kept constant. During the study, the participants sat in a comfortable armchair that allowed a relaxed and calm body and hand posture. The hand from which EDA was derived lay in a natural resting position on a soft surface. All recording sites were pretreated with ethyl alcohol (70%); the hands of the participants were additionally treated with lukewarm water beforehand. The positioning of the skull electrodes followed the international 10–20 system (Cooper et al., 1980; Jasper, 1958). After applying these electrodes and carefully fixing the electrode cables and plug connections without tension, the contact resistances (criterion: < 5 kΩ) and the physiological recordings were checked; if recording quality was acceptable by the experimenter, the first instruction was given.

### Instructions

The first instruction provided participants with general information about the course of events, the purpose of the study and the desired behavior in both phases of the study. The instruction for the *resting phase* followed immediately afterwards. Participants were asked to adopt a relaxed posture and keep their eyes open to ensure high-quality measurement of their physiological responses at rest.

The instruction for the *task phase* was given after the resting measurement and included the task sequence and the required behavior (see task). In addition, the participants were again asked to adopt a relaxed posture, keep their eyes open, and gaze forward toward a window in the cabin wall. Behind this window, equipped with a finely woven but transparent copper wire mesh was the monitor required for the task presentation. Between the first and second part of the task, a further but brief instruction was given explaining a changed requirement in the second part of the task.

### Measurement procedure

The physiological resting activity was measured after the corresponding instruction and a 3-min adaptation phase during a 5-min (stimulus-free) resting phase. The goal was to determine the electrodermal spontaneous activity, which is decisive for the classification of participants or rather the (quasi-experimental) variation of the independent variable lability. The actual task followed, but only after further instruction, task practice and (2-min) mental preparation for the task. This (renewed) instruction was necessary, because in the first part of the study, the participants should not yet know what to expect specifically in the second part. They were only informed in advance that they would face a reaction task in the main part of the study, which they did not have to worry about. Thus, the participants were honestly informed but not burdened with details that could have influenced the EDA during the resting phase. Continuous measurements were interrupted after the first 60 task trials to inform the participants about altered requirements in the second part of the task and to give them the opportunity to practice. The measurements were then continued after a further 2-min break to prepare for the task.

### Task

**Fig. 1:**
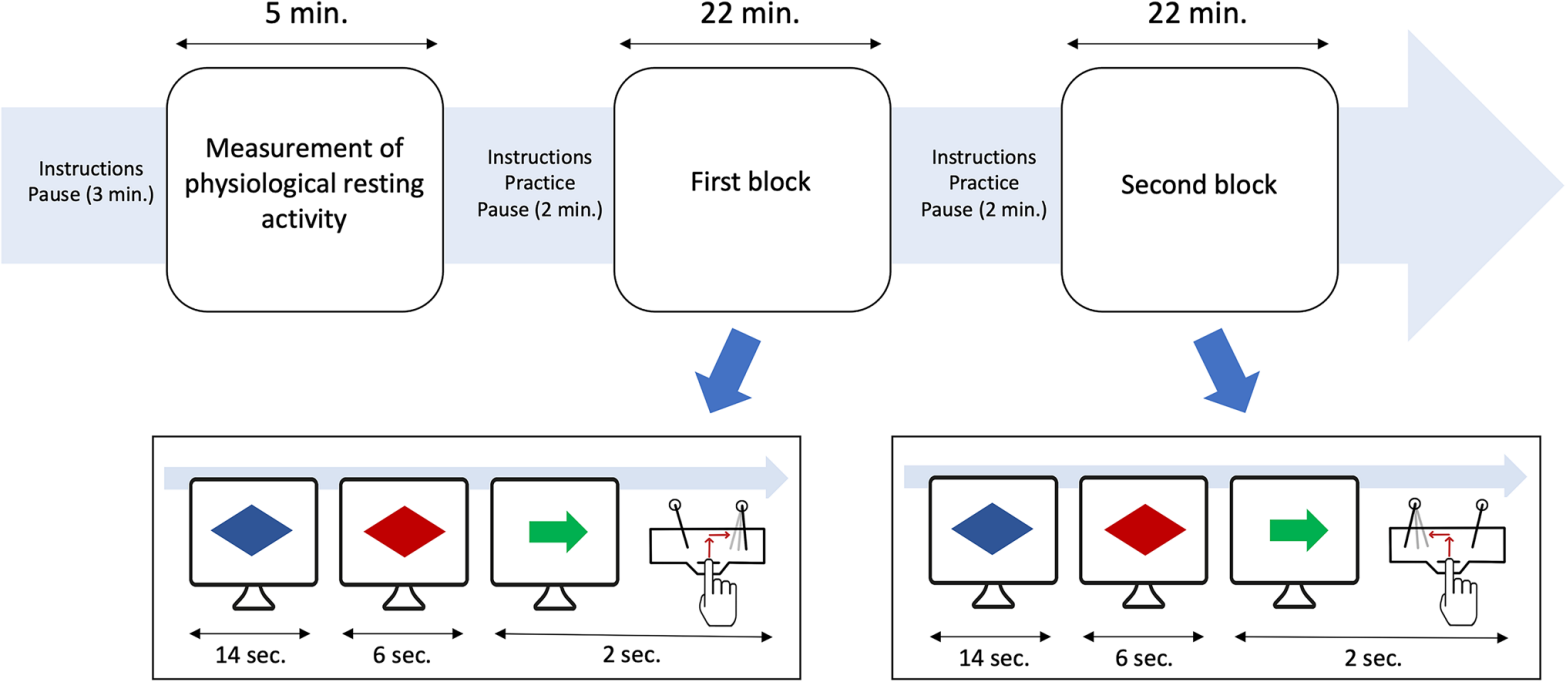
Study phases and task procedure. During the task, three stimuli appeared in chronological order. *Blue* diamond served as a sign for relaxation, *red* diamond as the signal (S1) for preparation for a response to be made immediately upon the appearance of the imperative stimulus (S2)—*green* arrow. Reaction time was measured as the time between onset of the S2 and releasing the RT button (middle button) in the 2-s response window. Two physiological indicators of preparation were measured at the end of the 6-s S1-S2 interval: CNV in the last and terminal HRD in the last 2 s. Note: The task changed from the first to the second block. In the first block, in response to the imperative stimulus, after releasing the RT button, the index finger of the right hand should be moved in the direction of the arrow and in the second block in the opposite direction

The task required *sustained attention* and coping with a change in demands in the second part of the task. Task *stimuli* were colored geometric shapes: a blue and red diamond and a green arrow, appearing in the middle of the monitor. The *blue diamond* served as a sign for relaxation (14 s), the *red diamond* as a signal (S1) for preparation (6 s) for a response to be made immediately upon the appearance of the *green arrow* (S2) (Fig. 1). Upon seeing the blue diamond, the *index finger of the right hand* was to rest on a microswitch (RT button). The appearance of the green arrow signaled to lift the index finger as quickly as possible and then to press one of two laterally (left and right) mounted levers corresponding to the direction of the arrow (2-s response window). No feedback on responses was provided. *Reaction time* was measured as the time between onset of the green arrow and *releasing* the RT button (middle button). Strategies such as guessing which stimulus would appear, counting the seconds elapsed, or rehearsing sections of the instructions were to be avoided.

To assess the effectiveness of the instructions, eight *practice trials* followed. During a break between the fourth and fifth trial, the participants were asked to repeat the instructions in their own words.

The *task* itself consisted of two identical blocks of 60 (22-s) trials each. The arrow direction varied pseudorandomly within the blocks. Each direction occurred 30 times. The participants were asked to follow the direction of the arrow with their finger in the *first block* and to react in the opposite direction in the *second block*. The instruction for the second block was given in a short break between the blocks. As before, participants had the opportunity to practice the task and were reminded to repeat the instruction during the break between the eight practice trials. Breaks, as well as the start and end of the study phases, were announced on the monitor. The two task blocks each started after a 2-min pause, during which the text “Task will begin shortly” was displayed.

In our study, a constant 6-s S1-S2 interval was chosen. It was intended to be long enough to measure the manifestation of the (terminal) CNV independently of the early (initial) CNV, and at the same time short enough to allow precise preparation for S2 and its demands. A relatively long period of time was chosen for the interval between the blue and red diamond to prevent both stimulus-induced and anticipatory influences on the baseline of the physiological measurements.

### Participant classification

To determine lability, the frequency of spontaneous fluctuations was measured in the 5-min resting phase before the task. Only phasic changes in the EDA that occurred without an apparent cause and exceeded an amplitude of 0.02 µS were considered. Individuals were then classified into the two lability groups based on the median (11.5) of the thus determined electrodermal resting activity.

### EDA

EDA was measured with the constant-voltage method (0.5 V; Fowles et al., 1981) on the thenar and hypothenar eminences of the left palm using sintered Ag/AgCl electrodes (1 cm ⌀) filled with 0.05 molar NaCl electrolyte (resolution: > 0.01 µS in the measuring range of 0–50 µS).

### ECG

The ECG was recorded with two sintered Ag/AgCl electrodes filled with commercially available electrode gel. They were attached to the manubrium of the sternum and to the left lower thorax (grounding at the right lower thorax). The continuously recorded raw signal was amplified 1000 times and digitized at 500 Hz. R-waves were then detected offline.

The R-wave intervals were converted into heart rate (unit: beats per minute [bpm]) and related to a real-time scale on a second-by-second basis according to the formula of Velden and Wölk (1987; see also Velden & Graham, 1988). To calculate the HR curve of a single 6-s preparation interval, the HR value of the last second before S1 was used as the subtrahend (baseline value) and the six subsequent HR values as minuends. The shape of the individuals’ HR curve was then determined by averaging over selected task trials.

### EEG

#### Recording

The EEG was recorded at six positions on the skull surface (F3, F4, C3, C4, P3, P4) with linked earlobes as reference. Chlorinated silver electrodes (on the skull) and sintered Ag/AgCl electrode clips (on the earlobes) were used for this purpose (effective electrode area in both cases: ≈ 1 cm^2^). An adhesive paste served as an electrolyte and for fixation. The bioelectric signals were amplified using a DC/AC amplifier with an additional low-noise differential preamplifier and a downstream variable filter unit. Brain activity was recorded with a gain of 10^4^, a time constant of 30 s, a low-pass filter of 30 Hz and a sampling rate of 125 Hz.

#### Artifact handling of recordings

A mathematical artifact correction was performed using the program developed by Gratton et al. (1983). A visual inspection was then performed to ensure successful correction. For this purpose, the corrected recordings were compared with the original recordings and the corresponding vertical and horizontal electrooculogram. Only recordings without apparent artifacts were selected for further data analysis. In addition, no blinks (> 150 µV) were allowed to occur at the time of S1 and S2 (for further selection criteria, see RT measurement). In most cases, more than two thirds of the task trials could be considered per individual and task block.

#### Analysis of event-related activity

The event-related brain activity of interest was calculated (in µV) from the continuously recorded and mathematically corrected surface potential fluctuations using the standard averaging procedure. The individuals’ potential changes were determined by averaging over selected trials as a mean course over seven seconds (from the onset of S1 to 1 s after S2) relative to a 1-s baseline (mean voltage value, measured in the second *immediately before* S1). Only the selected, artifact-free trials were used for this procedure.

### Measurement of dependent variables: RT, CNV, and tHRD

#### Reaction time

The *RT* was measured as the latency between the onset of S2 and the release of the RT button. Trials were excluded from the analysis if an incorrect response was given after the RT button was released or if no response was given during the 2-s response window. Trials in which a reaction occurred prematurely (before onset of S2) also were excluded. The total number of task trials excluded in this respect was negligible. These excluded trials were not included in the analysis or presentation of the CNV and tHRD.

#### CNV and HR deceleration

The *CNV* in the last second and the *tHRD* in the last 2 s before S2 were used as dependent variables to measure preparedness.

## Results

Although the number of usable trials varied between individuals, the same trials were always used within an individual to calculate the values of the three dependent variables. *Analyses of variance* were used for statistical hypothesis testing. For RT and tHRD, a two-way analysis was conducted with the factors *lability* and *block*, whereas for CNV, the additional factors *hemisphere* and *position* were included. The levels of the factors were: stable versus labile for *lability*, first versus second for *block*, left versus right for *hemisphere*, and frontal, central, and parietal for *position*. All main effects of lability and all interactions with it are reported. For interactions with the 3-level factor position, the epsilon correction (according to Greenhouse & Geisser, 1959) was performed, and the probability of the alpha error was adjusted. The corresponding correction factor epsilon (ε) is given in addition to the significance level achieved by the adjusted *p*-value. For the main effects of lability, the effect size *d* (Cohen, 1988) was additionally calculated (Table 1).

**Table 1:** Cohen’s *d* and statistics for the main effects of lability in the three dependent variables RT, CNV and tHRD

	Mean	SD	Maximum	Minimum	Cohen’s *d*
**RT**					
Stable	462.7917 ms	128.0561 ms	824 ms	254 ms	0.4607
Labile	410.0625 ms	098.9862 ms	674 ms	235 ms	
**CNV**					
Stable	-3.7723 µV	5.4235 µV	19.3670 µV	-20.7930 µV	0.3965
Labile	-6.0073 µV	5.8414 µV	11.5160 µV	-21.2660 µV	
**tHRD**					
Stable	-1.5801 bpm	1.3609 bpm	2.1684 bpm	-5.7609 bpm	0.7607
Labile	-3.0490 bpm	2.3676 bpm	2.3266 bpm	-9.2442 bpm	

Explanatory note to Cohen’s *d*: Effect sizes are usually interpreted as small (*d* = 0.2), medium (*d* = 0.5), and large (*d* = 0.8) based on benchmarks suggested by Cohen (1988). Bpm = beats per minute, CNV = contingent negative variation, ms = milliseconds, RT = reaction time, SD = standard deviation, tHRD = terminal heart rate deceleration, µV = microvolt.

### Reaction time

Labile individuals responded 52.7 ms faster than stable individuals in the task (*F*(1,46) = 4.57, *p* < 0.05; *d* = 0.46). When comparing the two task blocks, the RT showed an increase of 64.6 ms (*F*(1,46) = 84.20, *p* < 0.01). While the increase was slightly more pronounced in Stabiles (71.6 ms) than in Labiles (57.7 ms), this lability x block interaction was not significant (*F*(1,46) = 0.98, *p* = 0.33).

### HR deceleration and CNV

**Fig. 2:**
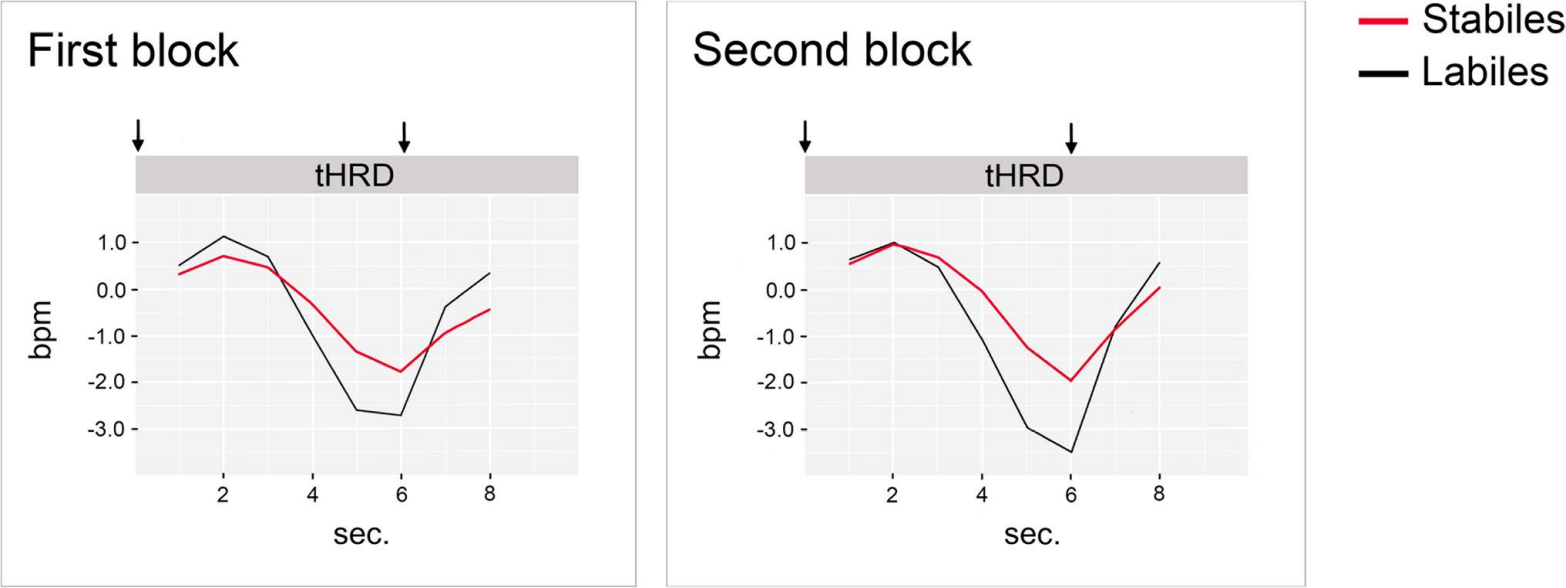
Change of HR (in bpm) in the 6-s preparation interval and in the subsequent 2-s response window of the task, depending on lability and task block. Arrows mark the beginning and end of the preparation interval. The heart rate baseline is represented by the value 0 on the vertical axis

The tHRD was significantly more pronounced in Labiles than in Stabiles (*F*(1,46) = 11.95, *p* < 0.01; *d* = 0.76), with a mean difference of 1.5 bpm (Fig. 2). The factor block had no significant effect (*F*(1,46) = 1.66, *p* = 0.20) on the tHRD. Likewise, no lability x block interaction occurred (*F*(1,46) = 1.50, *p* = 0.23).

**Fig. 3:**
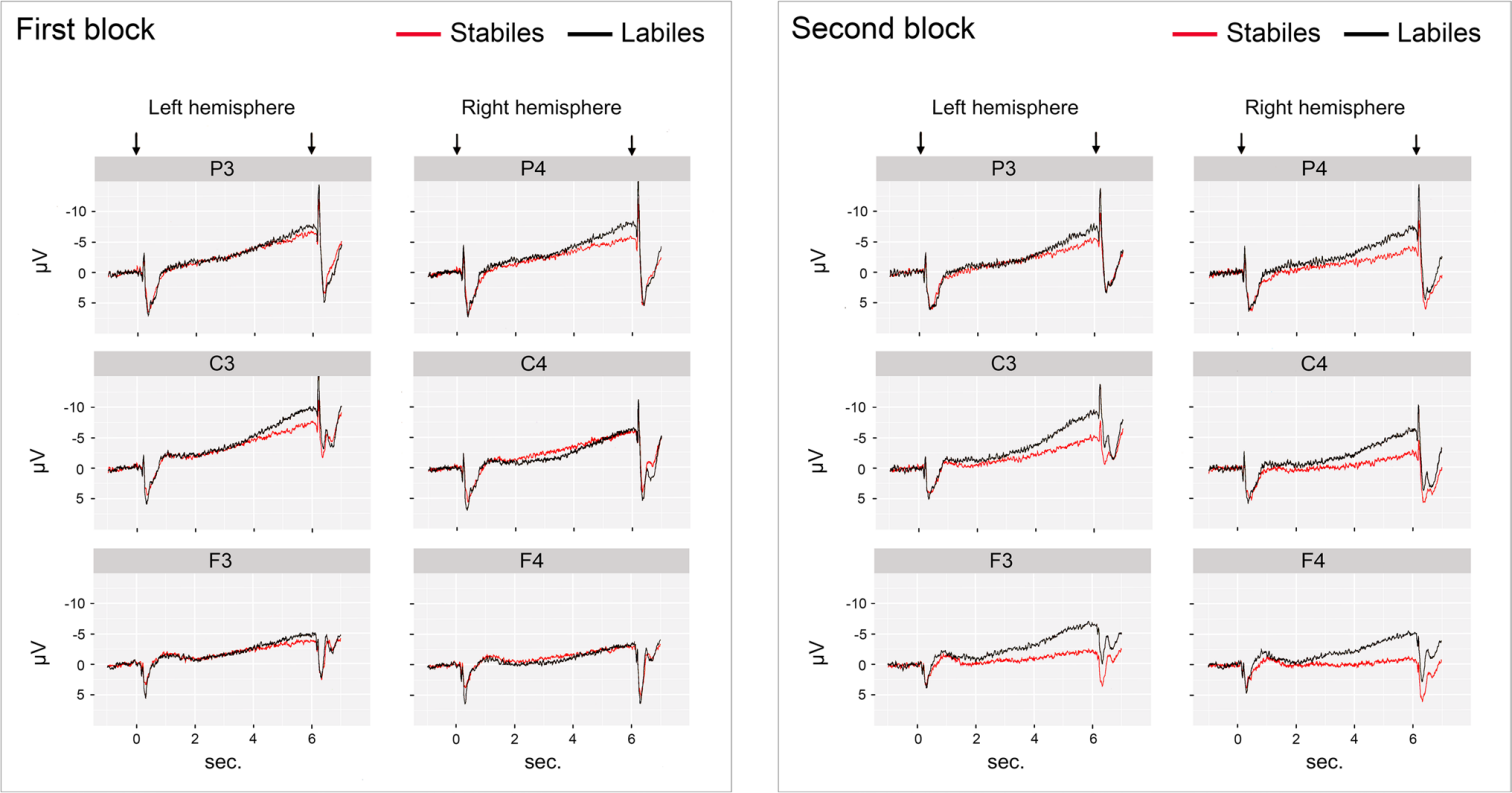
Voltage trace (in µV) during the task at six standardized recording sites on the skull surface in the second before S1 (baseline), during the subsequent preparation interval and in the second after S2, depending on lability and task block. Arrows indicate the onset of S1 and S2

The findings regarding the CNV were relatively complex because of the consideration of electrode positions and hemispheres (Fig. 3). Nevertheless, the expected main effect of lability was evident (*F*(1,46) = 4.67, *p* < 0.05; *d* = 0.40): Labiles showed a 2.2 µV stronger CNV than Stabiles.

While the CNV difference between Labiles and Stabiles was only significant in the second block (main effect of lability in block 2: *F*(1,46) = 8.05, *p* < 0.01), the lability x block interaction fell just short of the significance level (*F*(1,46) = 3.85, *p* = 0.06). The different localization of the phenomenon in the two task blocks is also striking. While the CNV difference in the first block can at best be seen graphically at the electrode positions C3 and P4, in the second block it was significant at five of the six locations, namely at F3 (*p* < 0.01), F4 (*p* < 0.01), C3 (*p* < 0.05), C4 (*p* < 0.05), and P4 (*p* < 0.05). Statistically, this was reflected in a significant interaction between lability, block and position (*F*(2,92) = 4.06, *p* < 0.05, ε = 0.81132).

It is noteworthy that the CNV of labile individuals did not decrease significantly across blocks and even slightly (0.05 < *p* < 0.10) increased frontally (frontal: + 1.7 µV, central: − 0.5 µV, parietal: − 0.7 µV) (*F*(2,46) = 5.76, *p* < 0.01, ε = 0.81512; for the block x position interaction among Labiles), while it decreased among stable individuals at all positions (*F*(1,23) = 5.21, *p* < 0.05), especially centrally (frontal: − 1.9 µV, *p* < 0.10, central: − 3.1 µV, *p* < 0.01, parietal: − 1.6 µV, *p* < 0.10) (*F*(2,46) = 4.61, *p* < 0.05, ε = 0.79081; for the block x position interaction among Stabiles). The main effect for block did not reach the significance level (*F*(1,46) = 2.96, *p* = 0.09), despite a slight attenuation of the CNV (by 1.0 µV).

Remarkable is further the *lateralization* of the CNV at the central positions (*F*(1,46) = 24.22, *p* < 0.01). Because of its left-sided dominance (− 6.95 µV on the left vs. − 4.52 µV on the right), it reflects the (right) body side of the reacting hand (Grünewald et al., 1979). The amplitude of the (terminal) CNV at position C3 thus appears to be suitable for testing lability effects in *motor preparation*. As expected from RT, there was a main effect of lability at this position (*F*(1,46) = 5.50, *p* < 0.05; with a mean difference of 3.0 µV), as well as an effect of block (*F*(1,46) = 7.00, *p* < 0.05), but no lability x block interaction—again despite slight differences between the lability groups, this time in the decrease of the CNV across the two task blocks. The decrease was somewhat stronger for Stabiles (2.5 µV) than for Labiles (0.8 µV). The effects on the left central CNV are similar to the effects on the RT. Nevertheless, the Pearson correlation between RT and CNV expected at C3 was moderate (*r* = 0.34, *p* < 0.05) and of the same order of magnitude as the Pearson correlation between RT and tHRD (*r* = 0.36, *p* < 0.05), which also was expected in the context of adequate motor preparation.

## Discussion

Electrodermally labile participants had shorter reaction times to the imperative stimulus compared with stable ones and showed in the final phase of preparation for the imperative stimulus a stronger response in both physiological indicators of preparation, namely in the (terminal) CNV and in the tHRD. Both hypotheses derived from earlier findings have proven to be useful. The average difference between the two groups was 52.7 ms in RT, 2.2 µV in CNV and 1.5 beats per minute in tHRD.

Thereby, both main questions of our study are addressed: (1) Labile and stable individuals differ not only in their RT, but also in both indicators of goal-oriented and adequate preparation, and (2) lability-dependent effects in forewarned RT tasks cannot be explained by differences in stimulus-driven (passive) processes alone. Rather, (active) processes that serve to adequately prepare for an imperative stimulus and are monitored by the supervisory attentional system (Norman & Shallice, 1986) also must be considered to explain them (Jennings & van der Molen, 2002). *Labiles not only react faster than Stabiles but also intentionally prepare themselves more appropriately for the imperative stimulus.*

Another issue can be tackled at the same time: Motor preparation appears to play a central role in the RT differences between labile and stable individuals, as the marked and statistically significant difference in the CNV at the left central recording site (C3) clearly indicates differences in the motor preparation for a response with the right hand. Labile individuals seem to find it easier to purposefully preactivate the reaction hand than stabile ones. In parallel with the lability effect at C3 and consistent with the explanation just given, the significant correlation between CNV and RT, well-known since Rebert and Tecce (1973), occurred at this recording site as well.

The lability effect in the tHRD can be interpreted analogously to the effect of lability in the CNV at C3. Labiles appear to prepare themselves motorically better than Stabiles for the rapid release of the RT button. This interpretation is supported by the significant correlation between tHRD and RT. However, it is important to note that despite an almost identical correlation as between CNV and RT at C3, the tHRD also may reflect peripheral physiological phenomena, such as selective inhibition of activities in the somatic musculature that are irrelevant or even disruptive to a rapid response of the index finger of the right hand (Obrist, 1976, 1981). However, based on their literature review, Jennings and van der Molen (2002) concluded that the central nervous inhibition of competing actions at the end of the preparation period can lead directly to a slowing of the HR. They emphasize that this is not just a motor, but a sensorimotor preparation for the anticipated task demands (Jennings & van der Molen, 2002, p. 444). It is likely that this also applies to our findings.

Nevertheless, the above interpretation of the lability effect in the CNV at C3 cannot be the only explanation for the main effect of lability in the CNV, because this effect cannot be attributed solely to the difference between Labiles and Stabiles at C3. Rather, the significant lability x block x position interaction points to a further phenomenon: a clear and significant reduction of the CNV in *Stabiles* across the two task blocks, which was strongest centrally, but can be seen graphically at all recording sites (see Fig. 3). Accordingly, the lability effect was only significant in the second block. Subsequent analyses revealed significant lability-dependent differences in the second block at all recording sites except for P3. For this extensive and apparently time-dependent phenomenon, there must be another explanation. *Most plausible is a weakness in the ability of Stabiles to maintain their task-related attention in the long term.* Such a phenomenon could be related to a decline in *alertness or vigilance*, as a certain time- or vigilance-dependent impairment of stable individuals, at least under monotonous conditions, has been known for a long time (Crider, 1979, 1993). It was once demonstrated in relation to vigilance in EEG (Burch & Greiner, 1960) and in behavior (Silverman et al., 1959; Survillo & Quilter, 1965). However, the fact that this impairment can also affect intentional processes that are under the higher-level control of the prefrontal supervisory attentional system is a novel finding (compare with the significant lability effects on left and right frontal CNV in the second block). Apparently, S1-S2 tasks must last long enough for lability-related effects to show up in RT and CNV.

In this context, the changed requirement in the second task block – not to react in the direction of the green arrow, but in the opposite direction—could play a crucial role, as well as the interplay of two frontoparietal networks involved in attention. According to Corbetta et al. (2008), the right dominant ventral frontoparietal network can interrupt the activity of the bilateral dorsal frontoparietal network in a stimulus-driven (bottom-up) manner. It can, however, be adjusted by the expectation- and goal-driven (top-down) dorsal frontoparietal network to filter out irrelevant sensory stimuli. In this way, it protects the dorsal network from distraction during focused attention. Both networks are thus involved in the reorientation of attention and the motor response to task-relevant sensory stimuli. Perhaps, a weakness of stable individuals lies in the fine-tuning of these two systems during changing task demands. It is known that stable individuals cope less well with changing tasks demands than labile individuals (Schell et al., 1988). However, their weakening vigilance is also likely to play a key role here. Corbetta et al. (2008) emphasize a functional relationship between the activity in the ventral network and the output of a neural system that is crucial for the vigilance and an external orientation of consciousness (Aston-Jones et al., 1991).

This system exerts its neuro-modulatory effect through release of norepinephrine and originates in the *locus coeruleus* (LC). Although the LC, or blue spot, is just a small cluster of neurons in the brainstem (in the dorsal region of the pons), its widely dispersed ascending projections have effects on quite a few brain functions (Aston-Jones & Cohen, 2005; Pineda, 1995, p. 144), of which it is considered crucial for vigilance (Aston-Jones & Bloom, 1981; Foote et al., 1983, p. 871; Aston-Jones et al., 1991). Additionally, brain regions involved in attention and motor function receive particularly dense LC innervation (Foote & Morrison, 1987).

The activity of this system may offer a neurophysiological explanation for the performance deficit of stable individuals: Their LC may be tonically (spontaneously) too inactive for a task that requires both focused attention and flexibility in motor response selection over an extended period. However, task-related (phasic) deficits may also be involved, as tonic activity of the LC (its baseline activity) influences the strength of its phasic activity (Aston-Jones et al., 1999).

In general, reduced tonic LC activity is, however, thought to promote task-related engagement and filtering of distractions (Aston-Jones & Cohen, 2005). Incidentally, this assumption is consistent with the notion put forward by Corbetta et al. (2008) that the ventral network is deactivated under high demands in a top-down fashion to limit the network's response to the narrow range of task-relevant stimuli. The key to understanding the (apparent) contradiction to our explanation of the performance deficit of stable individuals may therefore lie in the fact that a very narrow attentional focus caused by very low tonic LC activity is not conducive to flexibility in coping with changing task demands (Aston-Jones & Cohen, 2005, pp. 414, 420).

Neurophysiological findings suggest that the LC interacts closely with top-down influences from cortical systems (Aston-Jones & Cohen, 2005) to support task-related behavior: e.g., in signal detection tasks (approximately 200 ms) before the motor response and shortly after identifying the target stimulus (Aston-Jones et al., 1994; Rajkowski et al., 2004). The two modes of LC activity, in turn, adaptively adjust the responsivity of cortical target sites to facilitate or even disengage task-specific processes. In tasks that require focused attention for correct responses, LC neurons fire at a (task-dependent) moderate tonic frequency and selectively respond to task-relevant stimuli, but not to distractors, even if the latter differ only slightly from the target stimuli. Neither too low nor too high tonic LC activity seems to be adaptive to that end (Aston-Jones et al., 1999). Phasic LC activity is strongest and most persistent when the task is successfully completed; in phases of poor performance, it is significantly reduced or absent (Aston-Jones & Cohen, 2005, p. 413).

### Limitations

The resting median (11.5) of the spontaneous fluctuations was comparatively low, potentially due to a characteristic of the current study: there was considerably more time and opportunity for the subjects to adapt to the laboratory setting (partly because of the time-consuming electrode placement). Additionally, particular emphasis was placed on creating a trusting and relaxed atmosphere during the study to avoid cognitive and affective influences on the number of spontaneous fluctuations. Thus, the measured median likely reflects true resting activity. This could mean that a covariation of lability with relevant confounding variables such as anxiety or worry can be largely ruled out. Vossel and Zimmer (1990), for example, found a considerably higher median (28.5) in a sample of 590 male subjects under more usual but less controlled boundary conditions with the same recording device and electrodes, the same measurement duration, and identical criteria for the detection of spontaneous fluctuations.

If spontaneous electrodermal fluctuations at rest reflect baseline LC activity as we propose, the relationship between the number of these fluctuations and task performance may vary considerably depending on task—and boundary—conditions and may even exhibit an inverted U-shaped pattern across the total range of these fluctuations. Indeed, although the conditions for this pattern were not present in our study, according to the theory proposed by Aston-Jones and Cohen (2005), *reduced* tonic LC activity is expected to promote task-related engagement and distraction filtering as not only too low but also too high tonic LC activity is associated with weak performance. For example, when faced with distracting environmental stimuli, very labile individuals may have problems in challenging tasks. And this may be true simply because factors such as flustered state or exam nerves are likely to be associated with many spontaneous electrodermal fluctuations, high tonic LC activity and poor task performance. In contrast, relaxation was encouraged in our study, and the task was not even demanding. Instead, an outward orientation of consciousness was required, which should benefit individuals with a higher tonic LC activity, i.e., the more labile ones.

Skin conductance varies with the number of active palmar sweat glands (Fowles, 1986) and therefore depends on the total number of palmar sweat glands varying across individuals. This may influence the individual number of spontaneous fluctuations detected. For diagnostic purposes or for direct verification of the relationship between electrodermal spontaneous activity and LC activity, consideration of these genetic differences would be beneficial, although challenging. In addition, direct verification of the relationship would require invasive measurement of LC activity.

Only males took part in our study. It would be interesting to examine whether our findings are gender-specific. However, when examining women, one would have to consider cycle-dependent hormonal influences on electrodermal activity (Gómez-Amor et al., 1990; Sides et al., 2023).

Finally, yet importantly, while our research hypotheses were aligned with current research, the study was designed for heuristic purposes rather than to test deductive hypotheses because of the lack of a theory for adaptive control of goal-oriented preparation depending on electrodermal lability. Consequently, conclusions drawn from our data are inductive and subject to generalization issues when moving from data to theory. This concerns, for example, the position of the median, our task conditions and the male gender of our participants.

## Conclusions

The neuronal activity of the LC seems suited to explain electrodermal lability effects parsimoniously. From a theoretical point of view, our results as well as the already known lability effects in reaction and discrimination time, general alertness, and strength of the orienting response (Crider, 1993; Vossel, 1988) can presumably be attributed to differences in tonic and phasic LC activity. *The frequency of spontaneous electrodermal fluctuations at rest, used to determine electrodermal lability, therefore would be a peripheral, noninvasive and easily measurable indicator of baseline LC activity during wakefulness.*

This hypothesis is further supported by the parallelism between nonspecific electrodermal activity and tonic LC activity. Both the frequency of skin conductance fluctuations (Crider, 1993) and tonic LC activity (Aston-Jones et al., 1991; Steriade & McCarley, 1990) covary with wakefulness and an outward orientation of consciousness. Their baseline activity is low in a calm or relaxed waking state, even lower just before falling asleep. Both reach their low in paradoxical sleep—a phase of sensory isolation. In states such as stress or fear, both activities can be significantly elevated beyond normal levels. An exception is deep sleep, in which LC activity is very low, while (Koumans et al., 1968, p. 304) an unleashed storm of nonspecific electrodermal fluctuations can occur.

## Data Availability

None of the data or materials for the study reported here are available, and the study has not been preregistered.

## Footnotes

1 Because this supervisory attentional system is activated when conscious control is required (Norman & Shallice. 1986), it is involved in our study in yet another way: our RT task required attention to be sustained over an extended period.

## Code availability

Not appliable.
